# Supplementary figures and images for: Meteorological factors and tick density affect the dynamics of SFTS in jiangsu province, China
Source: PLoS Negl Trop Dis. 2022 May 9;16(5):e0010432. doi: 10.1371/journal.pntd.0010432 (PMC9119627; doi:10.1371/journal.pntd.0010432)

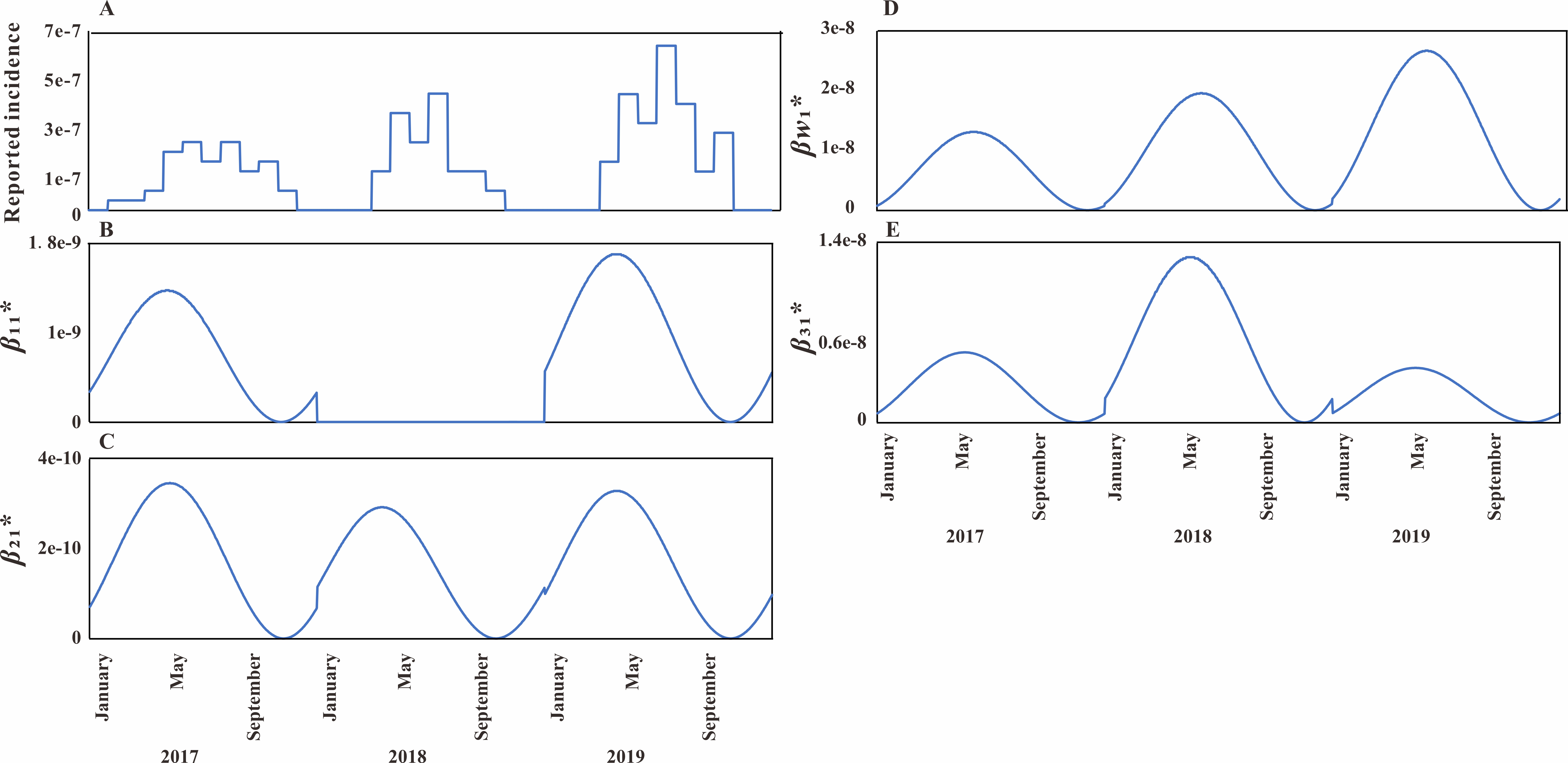

Supplement: S1 Fig — A: Reported incidence rate changes over time; B: Coefficient of environmental-to-human transmission of infection rate changes over time; βw1: Coefficient of environmental-to-human transmission of infection rate; C: Coefficient of human-to-human transmission of infection rate changes over time; β11: Coefficient of human-to-human transmission of infection rate; D: Coefficient of animal-to-human transmission of infection rate changes over time; β31: Coefficient of animal-to-human transmission of infection rate; E: Coefficient of tick-to-human transmission of infection rate changes over time; β21: Coefficient of tick-to-human transmission of infection rate. (TIF) [file pntd.0010432.s001.tif]

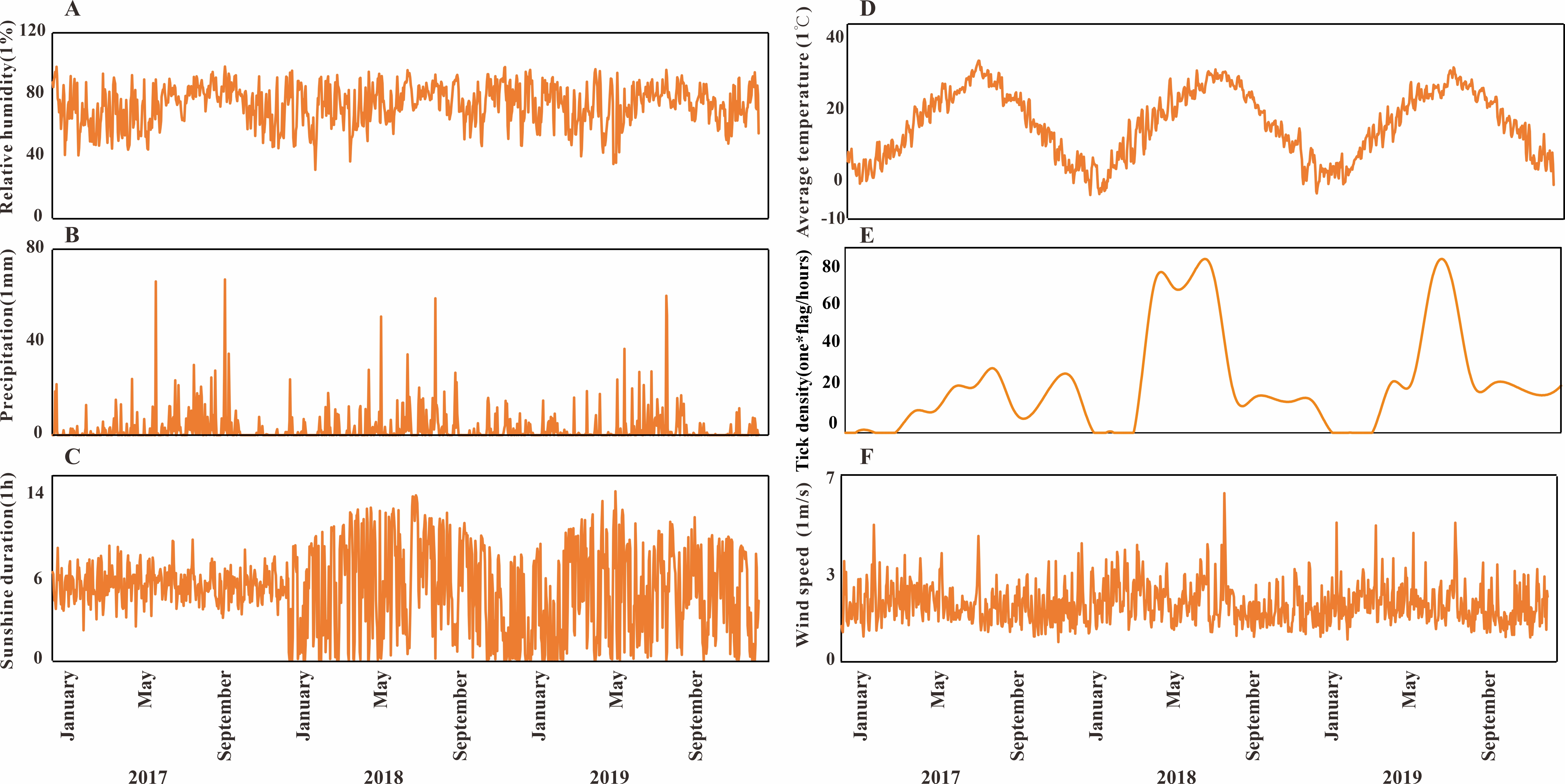

Supplement: S2 Fig — A: Relative humidity over time in Jiangsu Province; B: Precipitation changes over time; C: Sunshine duration changes over time; D: Average temperature changes over time; E: Tick density changes over time; F: Wind speed changes over time. (TIF) [file pntd.0010432.s002.tif]

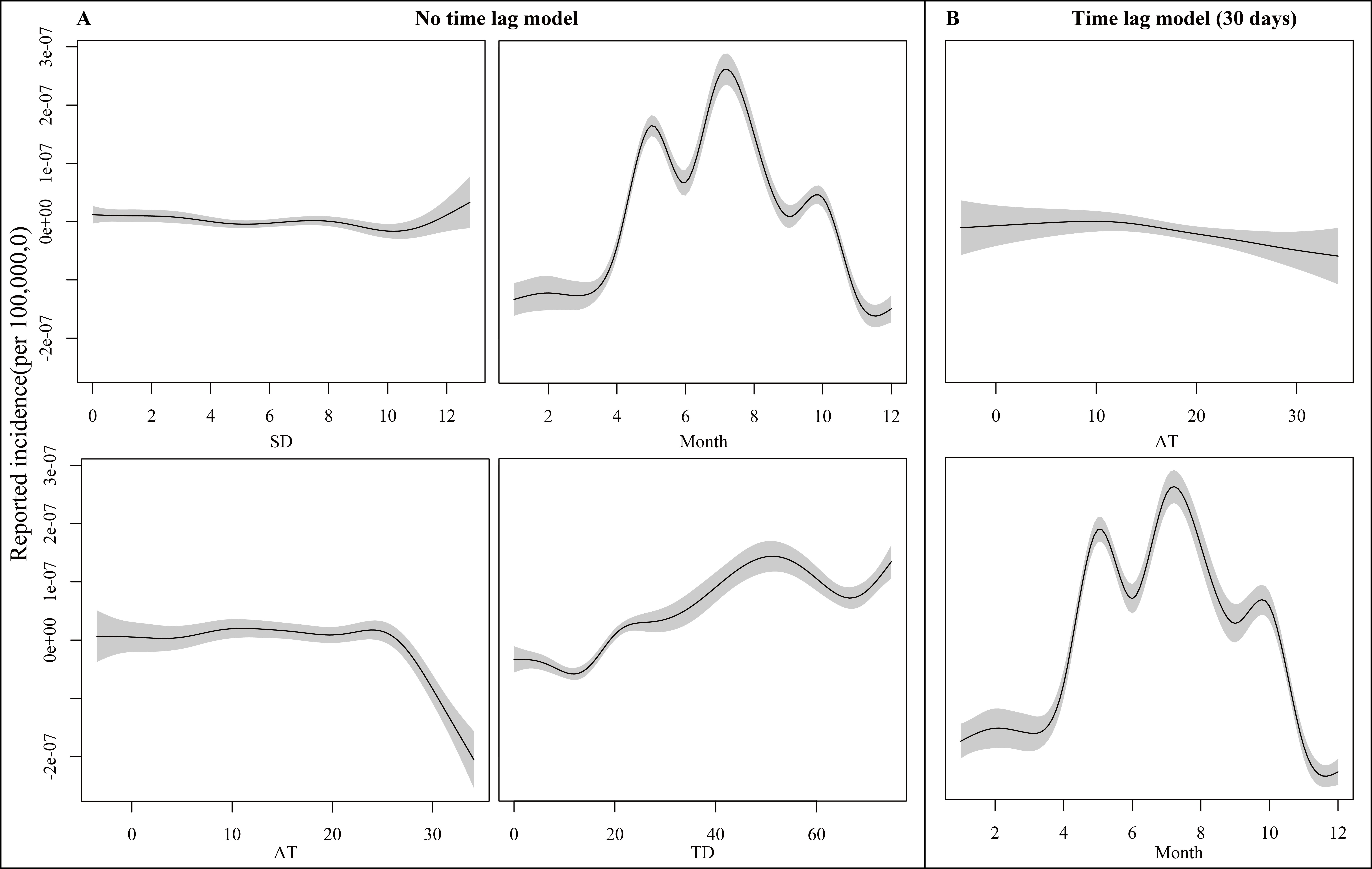

Supplement: S3 Fig — Part A: SFTS incidence with meteorological factors and tick density in no time lag GAM; Part B: SFTS incidence with meteorological factors and tick density in time lag GAM; SD = Sunshine duration; AT = Average temperature; WS = Wind speed; TD = Tick density. (TIF) [file pntd.0010432.s003.tif]

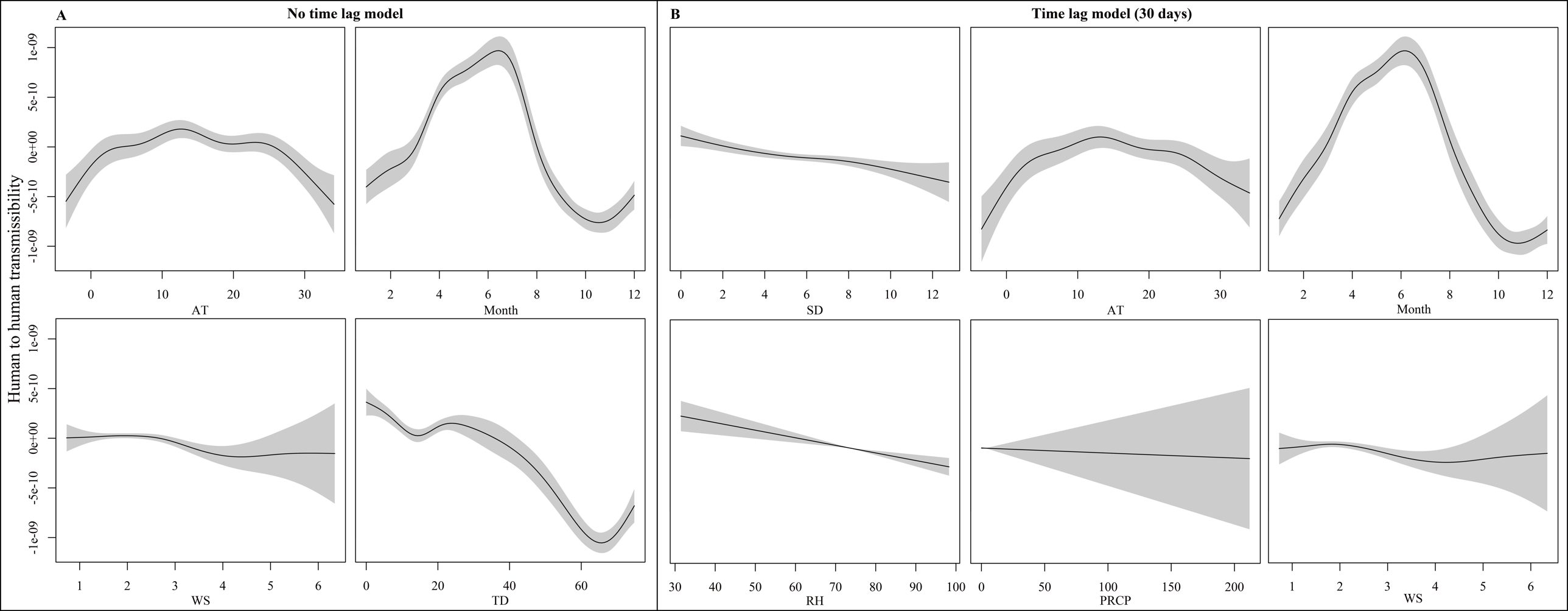

Supplement: S4 Fig — Part A: human-to-human transmissibility with meteorological factors and tick density in no time lag GAM; Part B: human-to-human transmissibility with meteorological factors and tick density in time lag GAM; SD = Sunshine duration; RH = Relative humidity; AT = Average temperature; PRCP = 24-hour precipitation; WS = Wind speed; TD = Tick density. (TIF) [file pntd.0010432.s004.tif]

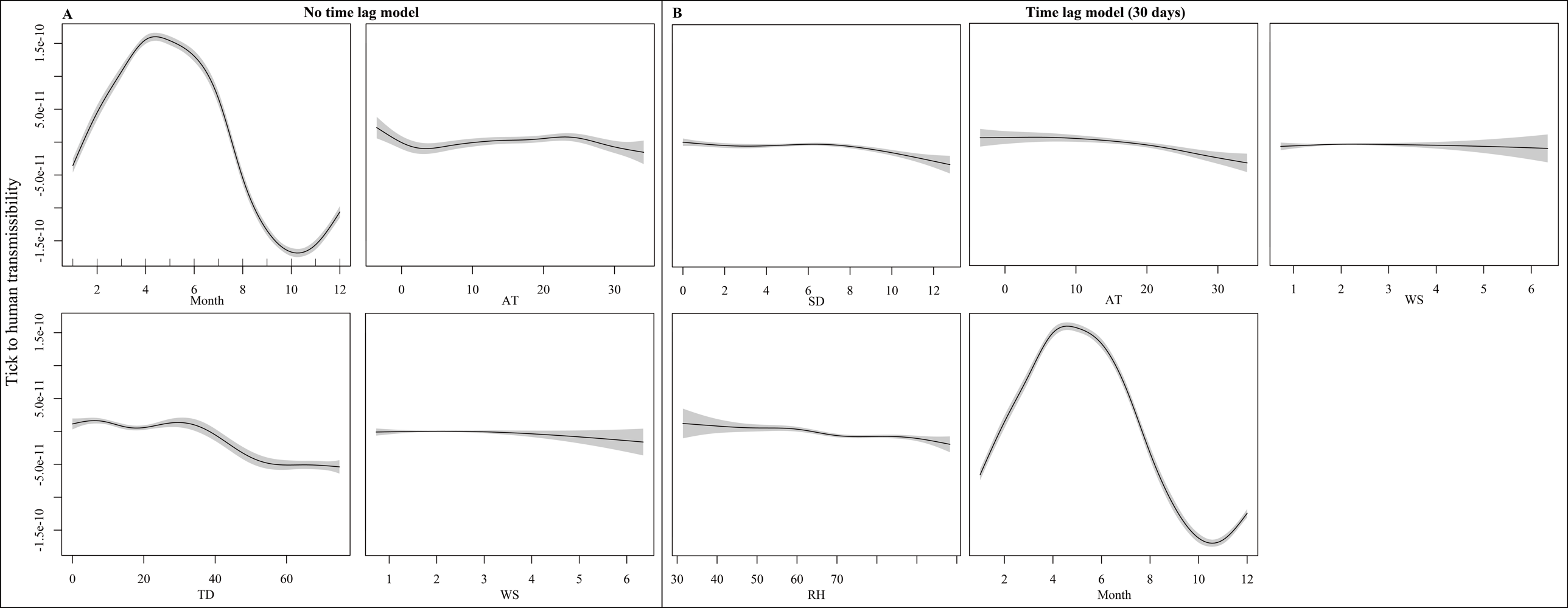

Supplement: S6 Fig — Part A: tick -to-human transmissibility with meteorological factors and tick density in no time lag GAM; Part B: tick-to-human transmissibility with meteorological factors and tick density in time lag GAM; SD = Sunshine duration; RH = Relative humidity; AT = Average temperature; PRCP = 24-hour precipitation; WS = Wind speed; TD = Tick density. (TIF) [file pntd.0010432.s006.tif]

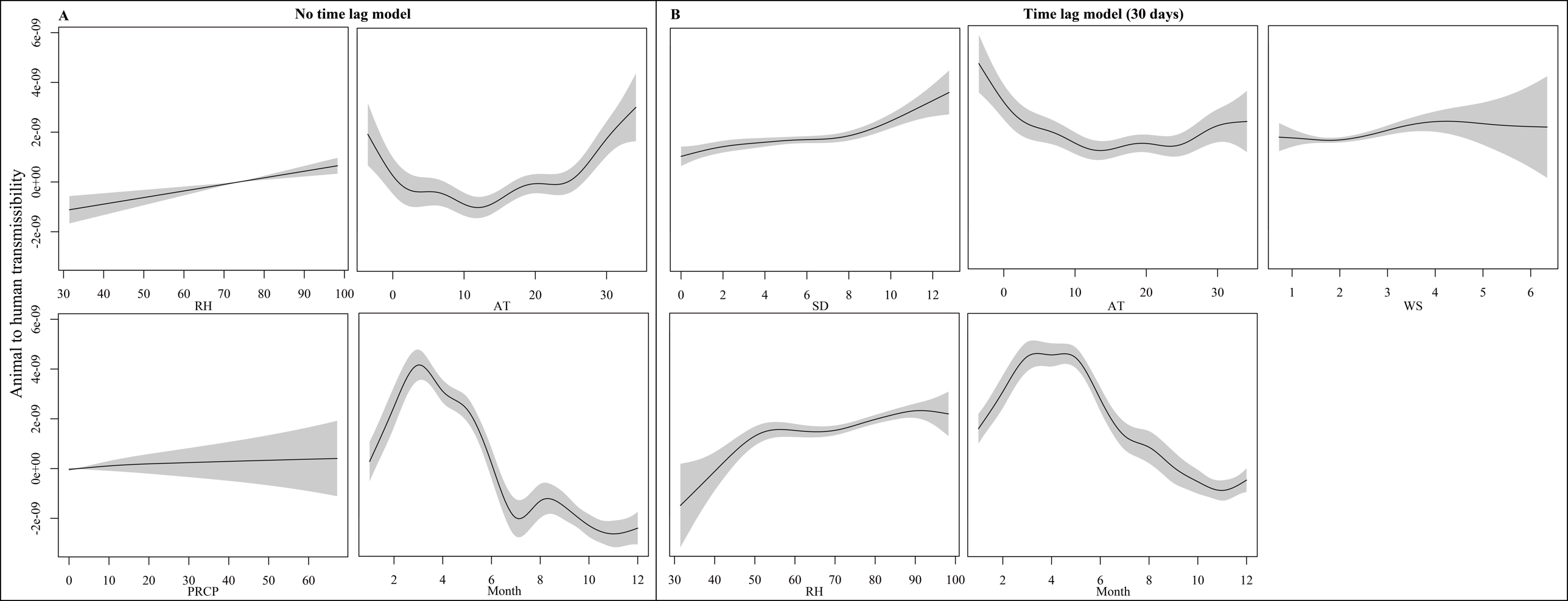

Supplement: S7 Fig — Part A: animal-to-human transmissibility with meteorological factors and tick density in no time lag GAM; Part B: animal-to-human transmissibility with meteorological factors and tick density in time lag GAM; SD = Sunshine duration; RH = Relative humidity; AT = Average temperature; PRCP = 24-hour precipitation; WS = Wind speed; TD = Tick density. (TIF) [file pntd.0010432.s007.tif]
