# Supplementary material for: Meteorological factors and tick density affect the dynamics of SFTS in jiangsu province, China
Source: PLoS Negl Trop Dis. 2022 May 9;16(5):e0010432. doi: 10.1371/journal.pntd.0010432 (PMC9119627; doi:10.1371/journal.pntd.0010432)
Supplement: Attachment — Submitted filename: Review comments and author responses.docx [file pntd.0010432.s009.docx]

Reviewer's Responses to Questions

**Key Review Criteria Required for Acceptance?**


As you describe the new analyses required for acceptance, please consider the following:


**Methods**


-Are the objectives of the study clearly articulated with a clear testable hypothesis stated?

-Is the study design appropriate to address the stated objectives?

-Is the population clearly described and appropriate for the hypothesis being tested?

-Is the sample size sufficient to ensure adequate power to address the hypothesis being tested?

-Were correct statistical analysis used to support conclusions?

-Are there concerns about ethical or regulatory requirements being met?


Reviewer #1: Bin Deng et al used multi-population and multi-route dynamic model aimed to explore whether the transmission routes of severe fever with thrombocytopenia syndrome (SFTS) will be affected by tick density and meteorological factors, and to explore the factors that affect the transmission of SFTS，However, the data and analysis in the article are not clear about the conclusions obtained.

***Author response:*** *Thank you so much for your useful comment. We apologize that we did not make the research design clear in its entirety. Based on your suggestions, we have redesigned the study idea in the Methods section and the Figure 1. First, we verified the four transmission routes of SFTS using the questionnaire of SFTS cases in Jiangsu province. We then used the established multi-population and multi-route dynamic model (MMDM) to calculate the infection rate coefficients of different transmission routes of SFTS in Jiangsu province, and also used the MMDM to simulate the effect of changing the effective contact rate of different transmission routes on the occurrence of SFTS. After that, we used the reported incidence and infection rate coefficients in Jiangsu Province from 17-19 years as dependent variables, and meteorological and tick surveillance data as independent variables to establish a generalized additive model (GAM), and used the Restricted maximum likelihood method to fit the model. We then used generalized cross validation to select the optimal model, and also used relevant data from Jiangsu Province in 2020 and Zhejiang Province in 2016 for model validation to obtain the most suitable GAM for elaborating SFTS transmission. Finally, we used the established GAM to predict the transmission of SFTS in Jiangsu Province in 2021 and the impact of extreme weather on SFTS. Our results suggest that the most effective intervention is to reduce human exposure to high-risk environments, and that different meteorological factors have different effects on the transmission pathways of SFTS, and that appropriate preventive and control measures should be taken for different changes in meteorological factors.* *After this revision, we believe that this study can elaborate the relationship between meteorological factors, tick density and SFTS transmission to some extent, and provide ideas for SFTS prevention and control.*

Reviewer #2: -Are the objectives of the study clearly articulated with a clear testable hypothesis stated?
Yes

-Is the study design appropriate to address the stated objectives?
Yes

-Is the population clearly described and appropriate for the hypothesis being tested?
Yes

-Is the sample size sufficient to ensure adequate power to address the hypothesis being tested?
Yes

-Were correct statistical analysis used to support conclusions?
Yes

-Are there concerns about ethical or regulatory requirements being met?
No ethical or regulatory concerns

Reviewer #3: (No Response)

--------------------

**Results**


-Does the analysis presented match the analysis plan?

-Are the results clearly and completely presented?

-Are the figures (Tables, Images) of sufficient quality for clarity?


Reviewer #1: the objective of this study is to obtain meteorological factors and tick density affect the infected dynamics of SFTS factors through modeling, not the affect about transmission routes.

***Author response:*** *Thank you so much for your valuable comment. We are sorry that we did not elaborate clearly the meaning and application of transmission routes in the model in our article. After revision, we added the definition of four transmission routes and restated the meaning of infection rate coefficients from line 222 to 231. In this study, we used the established transmission dynamics model to calculate the infection rate coefficients of different transmission routes of SFTS over time. The infection rate coefficient can be represented as a key parameter affecting the transmission routes. The generalized additive model was used to explore the relationship between the infection rate coefficients of each transmission routes and meteorological factors and tick surveillance density. The different degrees of influence of meteorological factors and tick density on the different infection rate coefficients were used to determine the key factors affecting the different infection rate coefficients and thus to elaborate the spread of SFTS.*
Reviewer #2: -Does the analysis presented match the analysis plan?
Yes

-Are the results clearly and completely presented?
No. The authors need to establish the generalizability of the model and make meaningful predictions using the model. The following major concerns need to be addressed:

1. GAMs are known to have the limitation of over-fitting. The authors need to calculate incidence and transmission of SFTS using an independent dataset, possibly from another province. If the model is found to be over-fitting, the authors need to more carefully select the independent meteorological variables since many of them were found to be significantly correlated with each other. They might also employ one of the dimensionality reduction techniques.
***Author response:*** *Thank you so much for your insightful comment. For GAM, the most important thing is the choice of the number of “knots” in curve fitting. When there are too many “knots”, it will cause overfitting problem, and too few “knots” will not show the relationship between variables better. Therefore, we have re-chosen the fitting method of the generalized additive model based on your suggestion. Under the premise of using the generalized cross-validation scores to select the model, we also use the Restricted maximum likelihood method to construct the model, which is more robust to the small sample size of SFTS. Not only that, we added the data of Jiangsu Province 2020 and Zhejiang Province 2016 to validate the model to prevent the overfitting problem you mentioned to the maximum extent. Before selecting meteorological factors and tick density, we also did correlation tests for the independent variables to avoid high correlation between independent variables that could affect the effect of GAM.* *We added Zhejiang Province 2016 and Jiangsu Province 2020 to the data collection for row 195. In the methods section, methodological supplements for infection rate coefficient calculation, intervention simulation, and GAM validation were added in rows 215, 236, and 279, respectively.*

2. The generalizability of the model need to be established by making predictions about national incidence and transmission rates employing the model.
***Author response:*** *Thank you so much for pointing this out. Yes, our previous build for GAM had some limitations. Based on your suggestion, we are also interested in making projections for SFTS nationwide. We are sorry to say that since a very important parameter, tick density, is needed in our model. However, it is difficult to obtain data because SFTS is a new infectious disease and a well-established tick surveillance system has not yet been established nationwide. Therefore, we could not make predictions for the whole country, but we tried to predict changes in tick density using meteorological factors by building a generalized additive model of ticks and meteorological factors. Therefore, we made predictions for Zhejiang Province using publicly available data for 2016 in line 502, and our results suggest that the model can be applied to places other than Jiangsu Province. In the next step, we will try to collect the national dataset and try to build the GAM that is applicable to predict the whole country.*

3. Although the authors have made recommendations about public health interventions for control and prevention of SFTS, they need to make predictions about how these interventions could impact SFTS incidence and transmission. Also they can make predictions about the possible impact of global climate change as well as regional natural calamities such as hurricanes, heavy rains or floods can have of SFTS incidence and transmission.
***Author response:*** *Thank you so much for your valuable comment.* *Yes, there are some problems with our previous recommendations on interventions. Based on your suggestion, we added effective exposure rates (θ) for different transmission routes in our multi-population and multi-route dynamic model (MMDM) and simulated θ from 10% to 100% through MMDM to reflect the effectiveness of controlling prevention and control for different transmission routes in line 335. Our results showed that controlling population exposure to high-risk environments is an important intervention to reduce the occurrence of SFTS, and we suggest that health education on SFTS prevention and control and avoidance of direct contact with the environment should be carried out for people living in high-risk environments. In the prediction of generalized additive model, two types of extreme weather, hurricane and drought, were simulated according to the geographical conditions and historical disaster information of Jiangsu Province. And the effects on SFTS were predicted during the duration of extreme weather, one month after the disaster, and one year after the disaster. Based on the results, we found that hurricanes would rapidly reduce the spread of SFTS and decrease the number of incidences in the short term, but drought had almost no effect on SFTS.*

-Are the figures (Tables, Images) of sufficient quality for clarity?
The figures need to be more self-explanatory. The labels are not legible in Figures 4, 5, 8 and 9.

Reviewer #3: (No Response)

--------------------

**Conclusions**


-Are the conclusions supported by the data presented?

-Are the limitations of analysis clearly described?

-Do the authors discuss how these data can be helpful to advance our understanding of the topic under study?

-Is public health relevance addressed?


Reviewer #1: some issues should be explained
1,Please explain the definitions of the four types of transmission: human-to-human transmission, tick-to-human transmission, environmental transmission and host animal transmission.

***Author response:*** *Thank you so much for your comment. Thanks for your reminding. We have added the definition of four transmission routes from line 222 to 231 of the revised manuscript. The full content is as follows: Based on the data from the 537 case questionnaire in Jiangsu province in 2018-2020, we defined human-to-human transmission as infected individuals without a history of tick bite but exposed to other patients; tick-to-human transmission as infected individuals with a history of tick bite; environmental-to-human transmission as infected individuals without a history of tick bite and without keeping or contacting animals but living in hilly areas; and animal-to-human transmission as infected individuals without a history of tick bite but who had contact with or kept animals.*

2,Usually, human-to-human transmission of SFTS is rare, and the transmission routes of the infected cases is not reported in this study.

***Author response:*** *Thank you so much for your valuable comment. Yes, human-to-human transmission of SFTS accounts for a small proportion of total cases. We re-collected questionnaires from 537 SFTS patients in Jiangsu province from 2018 to 2020 in line 200, and found that the number of cases of human-to-human infection had little change over the years, about 4-7%. Through the questionnaire, we found that the infection routes of human-to-human transmission cases were mainly attending funerals, taking care of patients, scrubbing patients' bodies and other direct contact with patients.*

Reviewer #2: -Are the conclusions supported by the data presented?
Yes

-Are the limitations of analysis clearly described?
Yes

-Do the authors discuss how these data can be helpful to advance our understanding of the topic under study?
No. The authors have not made significant efforts is making predictions about the changes in natural history of disease with global climate change and regional climate disasters

***Author response:*** *Thank you so much for your insightful comment. In the revision of the article, we added effective exposure rates for different transmission routes to the MMDM to assess the effectiveness of the intervention by varying effective exposure rates to simulate changes in mutual exposure rates between the environment, animals, humans, and ticks during the occurrence of SFTS in line 335. In addition, we assessed the short-, medium-, and long-term effects of extreme weather on disease using a validated generalized additive model of SFTS incidence changes in Jiangsu province under extreme weather conditions. We found that different weather extremes caused different effects, with hurricanes reducing SFTS incidence in the short term and having no long-term effects, but droughts having no effect on SFTS in line 502.*

-Is public health relevance addressed?
Yes. However, they do not employ their model to predict the impact of public health interventions could have on SFTS incidence and transmission, and recommend the most-effective interventions that can be implemented at the regional or national level.

***Author response:*** *Thank you so much for pointing this out. There are some problems with our previous recommendations on interventions. Based on your suggestion, we added effective exposure rates (θ) for different transmission routes in our multi-population and multi-route dynamic model (MMDM) and simulated θ from 10% to 100% through MMDM to reflect the effectiveness of controlling prevention and control for different transmission routes in line 236. Our results from line 344 to 349 showed that controlling population exposure to high-risk environments is an important intervention to reduce the occurrence of SFTS, and we suggest that health education on SFTS prevention and control and avoidance of direct contact with the environment should be carried out for people living in high-risk environments.*

Reviewer #3: (No Response)

--------------------

**Editorial and Data Presentation Modifications?**


Use this section for editorial suggestions as well as relatively minor modifications of existing data that would enhance clarity. If the only modifications needed are minor and/or editorial, you may wish to recommend “Minor Revision” or “Accept”.

Reviewer #1: Major revision

***Author response:*** *Thank you so much for your useful comment. We have made modification.*

Reviewer #2: The figures need to be more self-explanatory. The labels are not legible in Figures 4, 5, 8 and 9. The quality and presentation of the figures need significant improvement. The manuscript has to be proof-read by an English language expert.

***Author response:*** *Thank you so much for your useful comment. We have revised the manuscript for the revised images, and added clearer image annotations in the Figure legends section. Also, for language issues, we have invited native English experts to touch up.*

Reviewer #3: Correct term "a new Bunya virus" and " a new Bunia virus infection" (str. 71, p. 4; str 85, p 5). Severe fever with thrombocytopenia syndrome (SFTS) is an emerging infectious disease caused by Dabie bandavirus also known as the SFTS virus (Riboviria-Orthornavirae-Negarnaviricota-Polyploviricotina-Ellioviricetes-Bunyavirales-Phenuiviridae-Bandavirus-Dabie bandavirus)
***Author response:*** *Thank you so much for your useful comment. We have made modification in 123 line.*

--------------------

**Summary and General Comments**


Use this section to provide overall comments, discuss strengths/weaknesses of the study, novelty, significance, general execution and scholarship. You may also include additional comments for the author, including concerns about dual publication, research ethics, or publication ethics. If requesting major revision, please articulate the new experiments that are needed.

Reviewer #1: some issues should be explained
1,Please explain the definitions of the four types of transmission: human-to-human transmission, tick-to-human transmission, environmental transmission and host animal transmission.

***Author response:*** *Thank you so much for your comment. Thanks for your reminding. We have added the definition of four transmission routes in 225 line of the revised manuscript. The full content is as follows: Based on the data from the 537 case questionnaire in Jiangsu province in 2018-2020, we defined human-to-human transmission as infected individuals without a history of tick bite but exposed to other patients; tick-to-human transmission as infected individuals with a history of tick bite; environmental-to-human transmission as infected individuals without a history of tick bite and without keeping or contacting animals but living in hilly areas; and animal-to-human transmission as infected individuals without a history of tick bite but who had contact with or kept animals.*

2,Usually, human-to-human transmission of SFTS is rare, and the transmission routes of the infected cases is not reported in this study.

***Author response:*** *Thank you so much for your valuable comment. Yes, human-to-human transmission of SFTS accounts for a small proportion of total cases. We re-collected questionnaires from 537 SFTS patients in Jiangsu province from 2018 to 2020 in line 200, and found that the number of cases of human-to-human infection had little change over the years, about 4-7%. Through the questionnaire, we found that the infection routes of human-to-human transmission cases were mainly attending funerals, taking care of patients, scrubbing patients' bodies and other direct contact with patients.*

3, the objective of this study is to obtain meteorological factors and tick density affect the infected dynamics of SFTS factors through modeling, not the affect about transmission routes.

***Author response:*** *Thank you so much for your valuable comment. We are sorry that we did not elaborate clearly the meaning and application of transmission routes in the model in our article. After revision, we added the definition of four transmission routes and restated the meaning of infection rate coefficients from line 222 to 231. In this study, we used the established transmission dynamics model to calculate the infection rate coefficients of different transmission routes of SFTS over time. The infection rate coefficient can be represented as a key parameter affecting the transmission routes. The generalized additive model was used to explore the relationship between the infection rate coefficients of each transmission routes and meteorological factors and tick surveillance density. The different degrees of influence of meteorological factors and tick density on the different infection rate coefficients were used to determine the key factors affecting the different infection rate coefficients and thus to elaborate the spread of SFTS.*

4,Figure 4-9 is unclear should be load again
***Author response:*** *Thank you so much for your useful comment. We have revised the manuscript for the revised images, and added clearer image annotations in the Figure legends section. Also, for language issues, we have invited native English experts to touch up.*

Reviewer #2: The authors use a multi-population and multi-route dynamic model established previously to calculate the infection rate coefficients of various transmission routes of SFTS in Jiangsu Province of China, and a generalized additive model was established to further elaborate the influence of meteorological factors and tick density on SFTS incidence and transmission. The study is well-conducted, analyzed and interpreted. However, the readership and impact of the study will be greatly benefited if the following concerns are addressed.

Major comments
1. GAMs are known to have the limitation of over-fitting. The authors need to calculate incidence and transmission of SFTS using an independent dataset, possibly from another province. If the model is found to be over-fitting, the authors need to more carefully select the independent meteorological variables since many of them were found to be significantly correlated with each other. They might also employ one of the dimensionality reduction techniques.

***Author response:*** *Thank you so much for your insightful comment. For GAM, the most important thing is the choice of the number of “knots” in curve fitting. When there are too many “knots”, it will cause overfitting problem, and too few “knots” will not show the relationship between variables better. Therefore, we have re-chosen the fitting method of the generalized additive model based on your suggestion. Under the premise of using the generalized cross-validation scores to select the model, we also use the Restricted maximum likelihood method to construct the model, which is more robust to the small sample size of SFTS. Not only that, we added the data of Jiangsu Province 2020 and Zhejiang Province 2016 to validate the model to prevent the overfitting problem you mentioned to the maximum extent. Before selecting meteorological factors and tick density, we also did correlation tests for the independent variables to avoid high correlation between independent variables that could affect the effect of GAM.* *We added Zhejiang Province 2016 and Jiangsu Province 2020 to the data collection for row 195. In the methods section, methodological supplements for infection rate coefficient calculation, intervention simulation, and GAM validation were added in rows 215, 236, and 279, respectively.*

2. The generalizability of the model need to be established by making predictions about national incidence and transmission rates of SFTS employing the model.

***Author response:*** *Thank you so much for pointing this out. Yes, our previous build for GAM had some limitations. Based on your suggestion, we are also interested in making projections for SFTS nationwide. We are sorry to say that since a very important parameter, tick density, is needed in our model. However, it is difficult to obtain data because SFTS is a new infectious disease and a well-established tick surveillance system has not yet been established nationwide. Therefore, we could not make predictions for the whole country, but we tried to predict changes in tick density using meteorological factors by building a generalized additive model of ticks and meteorological factors. Therefore, we made predictions for Zhejiang Province using publicly available data for 2016 in line 502, and our results suggest that the model can be applied to places other than Jiangsu Province. In the next step, we will try to collect the national dataset and try to build the GAM that is applicable to predict the whole country.*

3. Although the authors have made recommendations about public health interventions for control and prevention of SFTS, they need to make predictions about how these interventions could impact SFTS incidence and transmission. Also they can make predictions about the possible impact of global climate change as well as regional natural calamities such as hurricanes, heavy rains or floods can have of SFTS incidence and transmission.
***Author response:*** *Thank you so much for your valuable comment. Yes, there are some problems with our previous recommendations on interventions. Based on your suggestion, we added effective exposure rates (θ) for different transmission routes in our multi-population and multi-route dynamic model (MMDM) and simulated θ from 10% to 100% through MMDM to reflect the effectiveness of controlling prevention and control for different transmission routes in line 335. Our results showed that controlling population exposure to high-risk environments is an important intervention to reduce the occurrence of SFTS, and we suggest that health education on SFTS prevention and control and avoidance of direct contact with the environment should be carried out for people living in high-risk environments. In the prediction of generalized additive model, two types of extreme weather, hurricane and drought, were simulated according to the geographical conditions and historical disaster information of Jiangsu Province. And the effects on SFTS were predicted during the duration of extreme weather, one month after the disaster, and one year after the disaster. Based on the results, we found that hurricanes would rapidly reduce the spread of SFTS and decrease the number of incidences in the short term, but drought had almost no effect on SFTS.*

Minor Comments
1. The figures need to be more self-explanatory.
2. The labels are not legible in Figures 4, 5, 8 and 9.
3. The quality and presentation of the figures need significant improvement.
4. The manuscript has to be proof-read by an English language expert.

***Author response:*** *Thank you so much for your useful comment. We have revised the manuscript for all the revised images, and added clearer image annotations in the Figure legends section. Also, for language issues, we have invited native English experts to touch up.*

Reviewer #3: The paper by Deng et al. describes the influence of different meteorological factors (wind speed, duration of sunshine, average temperature, amount of precipitation, atmospheric pressure and relative humidity) and ticks density on the incidence of f severe fever with thrombocytopenia syndrome (SFTS).
The relationship between all meteorological factors, tick density and was described by statistical method (Spearmen test). The relationship between SFTS incidence rates, SFTS transmission rate and different meteorological indicators and ticks density was assessed by constructing generalized additive models (GAMs) in in R 3.2.3. The choice of the most suitable model was determined based on the generalized cross-validation score (GCV).
It has been shown that the greatest influence on the incidence rate is exerted by the sunshine duration, temperature and relative humidity.
The paper is well illustrated, the conclusions are supported by the obtained results.
The new data obtained in the work can be used to predict the SFTS incidence.

Changes should be made to improve the manuscript:

1. Describe the epidemiology of SFTS in China, incl. describe the main route of human infection in Jiangsu Province from 2017 to 2019. When constructing models for various mechanisms of infection (β1, β21, β31 and βw1), were all cases of human disease taken into account or only with a certain route of infection? Explain in more detail the epidemiological meaning of transmission routes β1, β21, β31 and βw1.

***Author response:*** *Thank you so much for your useful comment. Due to data limitations, we have described the epidemiological characteristics of SFTS in Jiangsu Province from 2017 to 2019 in a previously published article. Therefore, the location and seasonal characteristics of confirmed cases in Jiangsu province from 2017 to 2019 were described in this paper. In view of infection routes of SFTS cases in Jiangsu Province, we collected questionnaires of 537 confirmed cases from 2018 to 2020, and added changes of infection routes in Jiangsu province in the past three years in line XX, and detailed information is also added in Table X. When constructing the multi-population and multi-route model, we considered all confirmed cases and calculated the number of possible cases infected by each route based on the proportion of previous infection routes, so as to build the model. In line XX, we add the definitions of four transmission routes and reiterate the definition of the infection rate coefficient, which can be expressed as a key parameter affecting the transmission route. In the transmission dynamics model, the fatality rate, birth rate, number of cases and other parameters can be obtained through literature or data. However, as a complex tick-borne disease, SFTS cannot calculate R0 to estimate the transmissibility like COVID-19. Therefore, the infection rate between different species can only be calculated by model, we can think that β1, β21, β31 and β W1 can represent the transmissibility of the four transmission routes to some extent.*

2.Describe what species of ticks are vectors of SFTS virus in China. Was the ticks species composition taken into account when assessing the number of ticks and their effect on the incidence rate?

***Author response:*** *Thank you so much for your useful comment. According to previous studies, Haemaphysalis longicornis is the dominant tick in the transmission of SFTS in Asia, accounting for about 98% of all cases. Unfortunately, as SFTS is a newly emerging infectious disease, it has not been listed as a statutory infectious disease in China, so many disease surveillance systems are relatively perfect. Tick monitoring has not been carried out in most areas, and the identification of tick species is weak. Therefore, tick species have not been considered in our transmission dynamics model, but we believe that the ability of different tick species to transmit viruses is different, which is also one of the important parameters for establishing transmission dynamics. In subsequent studies, we will further consider the impact of different ticks, providing ideas for the prevention and control of SFTS.*

--------------------

PLOS authors have the option to publish the peer review history of their article ([what does this mean?](https://journals.plos.org/plosntds/s/editorial-and-peer-review-process#loc-peer-review-history)). If published, this will include your full peer review and any attached files.


If you choose “no”, your identity will remain anonymous but your review may still be made public.


**Do you want your identity to be public for this peer review?** For information about this choice, including consent withdrawal, please see our [Privacy Policy](https://www.plos.org/privacy-policy).

Reviewer #1: No

Reviewer #2: No

Reviewer #3: No


Figure Files:
While revising your submission, please upload your figure files to the Preflight Analysis and Conversion Engine (PACE) digital diagnostic tool, https://pacev2.apexcovantage.com. PACE helps ensure that figures meet PLOS requirements. To use PACE, you must first register as a user. Then, login and navigate to the UPLOAD tab, where you will find detailed instructions on how to use the tool. If you encounter any issues or have any questions when using PACE, please email us at figures@plos.org.
 
Data Requirements:
Please note that, as a condition of publication, PLOS' data policy requires that you make available all data used to draw the conclusions outlined in your manuscript. Data must be deposited in an appropriate repository, included within the body of the manuscript, or uploaded as supporting information. This includes all numerical values that were used to generate graphs, histograms etc.. For an example see here: http://www.plosbiology.org/article/info%3Adoi%2F10.1371%2Fjournal.pbio.1001908#s5.
 
Reproducibility:
To enhance the reproducibility of your results, we recommend that you deposit your laboratory protocols in protocols.io, where a protocol can be assigned its own identifier (DOI) such that it can be cited independently in the future. Additionally, PLOS ONE offers an option to publish peer-reviewed clinical study protocols. Read more information on sharing protocols at https://plos.org/protocols?utm_medium=editorial-email&utm_source=authorletters&utm_campaign=protocols

__________________________________________________
In compliance with data protection regulations, you may request that we remove your personal registration details at any time. (Use the following URL: https://www.editorialmanager.com/pntd/login.asp?a=r). Please contact the publication office if you have any questions.
