# Supplementary material for: Meteorological factors and tick density affect the dynamics of SFTS in jiangsu province, China
Source: PLoS Negl Trop Dis. 2022 May 9;16(5):e0010432. doi: 10.1371/journal.pntd.0010432 (PMC9119627; doi:10.1371/journal.pntd.0010432)
Supplement: Attachment — Submitted filename: Reviewer attachments_PNTD_10.04.2022.docx [file pntd.0010432.s010.docx]

Anna Volynkina,

Head of Laboratory of Viral Infections

Diagnostic,

Stavropol Research Antiplague Institute,

355035, Stavropol, Russian Federation

10.04.2022

Dear Dr. Mohamed Gomaa Kamel,

The paper by Deng et al. describes the influence of different meteorological factors (wind speed, duration of sunshine, average temperature, amount of precipitation, atmospheric pressure and relative humidity) and ticks density on the incidence of f severe fever with thrombocytopenia syndrome (SFTS).

The relationship between all meteorological factors, tick density and was described by statistical method (Spearmen test). The relationship between SFTS incidence rates, SFTS transmission rate and different meteorological indicators and ticks density was assessed by constructing generalized additive models (GAMs) in in R 3.2.3. The choice of the most suitable model was determined based on the generalized cross-validation score (GCV).

It has been shown that the greatest influence on the incidence rate is exerted by the sunshine duration, temperature and relative humidity.

The paper is well illustrated, the conclusions are supported by the obtained results. The new data obtained in the work can be used to predict the SFTS incidence.

The manuscript has improved a lot after revision. Authors added detailed information about SFTS human infection routes in Jiangsu province in 2018-2020 and explained the definitions of the four types of transmission: human-to-human transmission, tick-to-human transmission, environmental transmission and host animal transmission.

The authors have addressed all of my comments. I now would like to support the publication of the current form of the manuscript.

Kind regards,

Anna Volynkina
